# Microbial Morphology and Motility as Biosignatures for Outer Planet Missions

**DOI:** 10.1089/ast.2015.1376

**Published:** 2016-10-01

**Authors:** Jay Nadeau, Chris Lindensmith, Jody W. Deming, Vicente I. Fernandez, Roman Stocker

**Affiliations:** ^1^ GALCIT, California Institute of Technology, Pasadena, California.; ^2^ Jet Propulsion Laboratory, California Institute of Technology, Pasadena, California.; ^3^ Department of Biological Oceanography, University of Washington, Seattle, Washington.; ^4^ Department of Civil and Environmental Engineering, Massachusetts Institute of Technology, Cambridge, Massachusetts.

## Abstract

Meaningful motion is an unambiguous biosignature, but because life in the Solar System is most likely to be microbial, the question is whether such motion may be detected effectively on the micrometer scale. Recent results on microbial motility in various Earth environments have provided insight into the physics and biology that determine whether and how microorganisms as small as bacteria and archaea swim, under which conditions, and at which speeds. These discoveries have not yet been reviewed in an astrobiological context. This paper discusses these findings in the context of Earth analog environments and environments expected to be encountered in the outer Solar System, particularly the jovian and saturnian moons. We also review the imaging technologies capable of recording motility of submicrometer-sized organisms and discuss how an instrument would interface with several types of sample-collection strategies. Key Words: *In situ* measurement—Biosignatures—Microbiology—Europa—Ice. Astrobiology 16, 755–774.

## 1. Microscopic Imaging for Life Detection: The Concept

Motility was one of the first accepted biosignatures. When Leeuwenhoek first observed bacteria and protozoa in the 17^th^ century using single-lens microscopes, he knew they were alive because of their motion: “[N]o more pleasant sight has ever yet come before my eye than these many thousands of living creatures, seen all alive in a little drop of water, moving among one another, each several creature having its own proper motion” (Macnab, 1999). He was able to resolve cilia, calling them “diverse incredibly thin feet, or little legs” (Dobell and Leeuwenhoek, 1932). Meaningful, directed motion, clearly distinguished from Brownian motion and diffusion, is an unambiguous biosignature that makes no assumptions about the chemical composition of the organisms under study. As early as 1966, “Motion of a type that would not be expected for non-living systems” was suggested as a biosignature for Mars missions (Glaser, 1966).

**FIG. 1.**
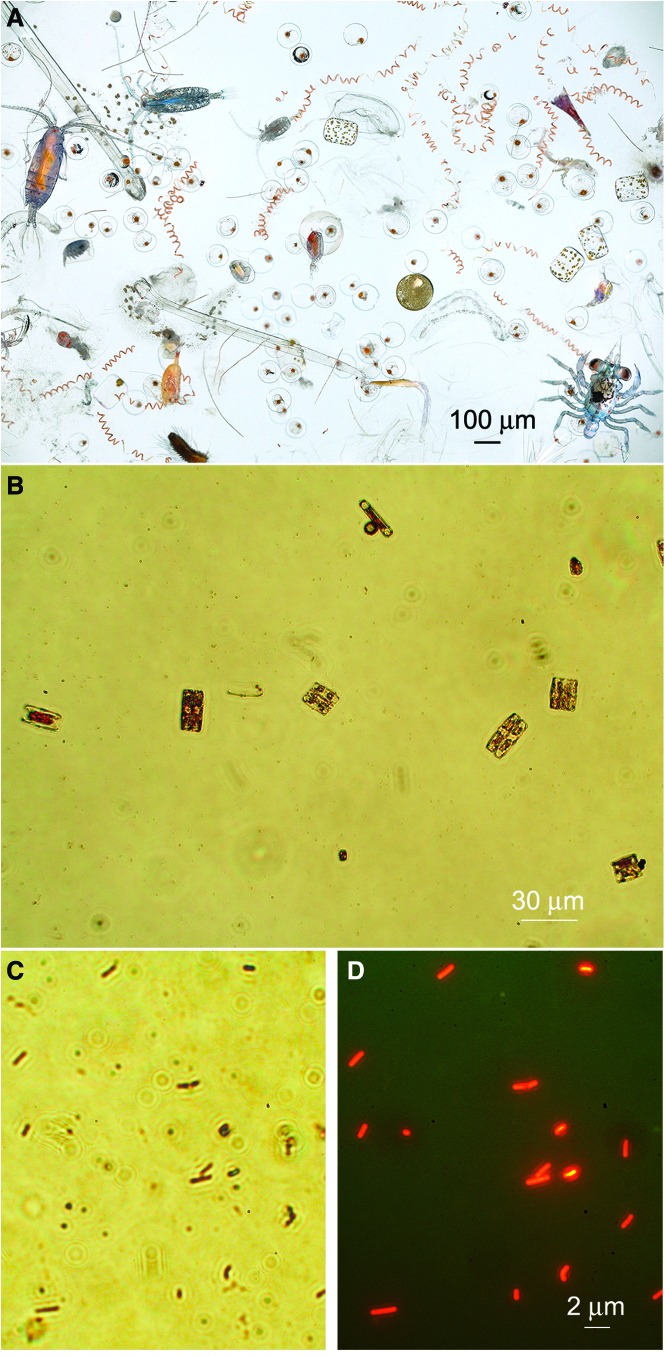
Microscopic morphology as a biosignature. (**A**) Marine microplankton, part of the contents of one dip of a hand net, photographed on board the NOAA Ship Oscar Elton Sette off Kona, September 20, 2006. The image contains diverse planktonic organisms, ranging from photosynthetic cyanobacteria and diatoms to many different types of zooplankton, including both holoplankton (permanent residents of the plankton) and meroplankton (temporary residents of the plankton, *e.g.*, fish eggs, crab larvae, worm larvae) (image courtesy David Liittschwager). (**B**) Low-magnification image of brine collected from sea ice near Nuuk, Greenland, from a sackhole in spring 2015. On this scale, examples of diatoms can be seen. (**C**) High-magnification bright-field image of Greenland brine showing numerous prokaryotes. (**D**) The same image from (C) stained with acridine orange and imaged with epifluorescence microscopy. Because staining labels DNA, this technique permits distinguishing bacteria from debris. The combination of multiple dyes, such as acridine orange and DAPI, can further remove ambiguity caused by nonspecific adherence of dyes to minerals.

As exploration of our solar system widens to include more likely habitats for extant microbial life than the ancient, dry terrain of Mars, direct imaging of microorganisms becomes more and more attractive as a target, one that is beginning to attract interest in the astrobiology community. For both motion and morphology, direct microscopic observation remains one of the best methods for detecting prokaryotes, the non-nucleated and smallest organisms on Earth (Kepner and Pratt, 1994). In eutrophic environments, simple observation of nucleated, compartmentalized, or even multicellular structures (eukaryotes) is usually sufficient to establish the presence of life (Fig. 1A). However, in oligotrophic environments, the organisms are less abundant, less diverse morphologically, and smaller. Specific cell dyes are often needed to distinguish the smallest of the organisms from debris, placing greater demands upon the detection instrument (Fig. 1B–1D).

**FIG. 2.**
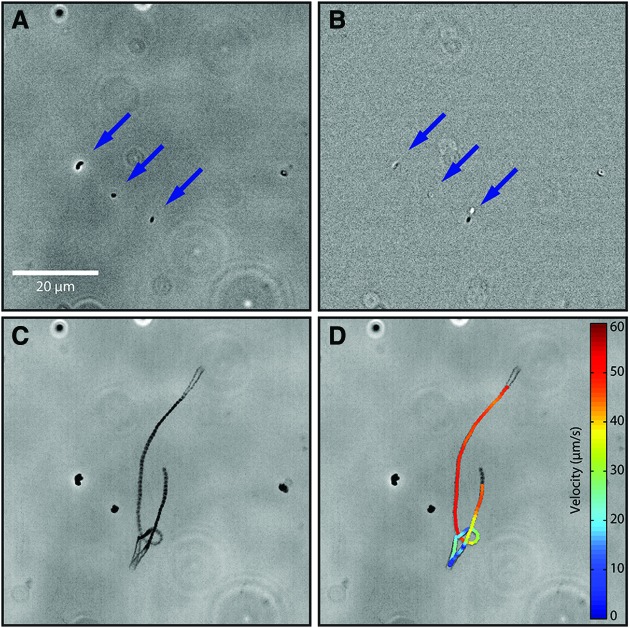
Motility as a biosignature. Microbial motility can be readily distinguished from the motion of passive particles. (**A**) A phase-contrast image (20× objective) of a liquid sample containing both passive particles (1 μm beads) and motile microorganisms (*Vibrio alginolyticus* bacteria). Three of these are identified by the blue arrows. From the still image alone, passive particles cannot be distinguished from motile microorganisms. (**B**) Time difference between two frames 0.06 s apart from the same phase-contrast video from which (A) was taken. Note the lower “particle,” which shows up as a white and a black speck due to its significant translation in the time interval of the frame difference. The other two particles hardly move, and the difference thus largely cancels out. (**C**) Minimum-intensity projection over the duration of the video (3.1 s). This image was constructed by taking the minimum value of each pixel over the duration of the video. The motile microorganism is clearly distinguished from the nonmotile particles that are solely under the influence of Brownian motion. (**D**) Particle tracking routines that extract the location of each “particle” in each frame and then connect locations among frames, revealing not only the trajectory of a microorganism but also its instantaneous planar speed (color bar), permitting the study of its behavior. The linear nature of much of the trajectory and most importantly its velocity allows one to confidently rule out Brownian motion as the source of the movement.

The detection of motility requires less spatial resolution than is required to unambiguously recognize single cells. Organisms that appear ambiguous under still images, especially when subresolved, are clearly alive under time-lapse imaging, with motion distinct in trajectory and velocity from Brownian motion (Fig. 2 and Supplementary Video S1; Supplementary Videos are available at www.liebertonline.com/ast). The most direct metric for differentiating between passive particles and motile cells is diffusivity, characterizing the Brownian motion of nonmotile particles. From the Stokes-Einstein equation, $D = \displaystyle { \frac { { k_ { \rm { B } } } T }  { 6\pi \eta r } }$, the diffusivity of a spherical passive particle depends on the radius of the particle (*r*) and the environmental temperature (*T*) and viscosity (η). The diffusivity of an individual can be estimated through the mean-square displacement 〈*x*^2^〉 = 2*Dt*, where *t* is time. For a 1 μm sphere in water, this gives a displacement of 0.01 μm s^−1^, compared with swimming speeds of 10–100 μm s^−1^.

For particles that are subresolved, we can use the resolution limit of the microscope to estimate an upper limit for the diffusivity to use in the diffusion equation. The observed size can be used when the particles are large enough to obtain resolved images. Strong currents or turbulence may generate ambiguity and will degrade estimates of diffusivity, making imaging in a relatively quiescent environment such as a small container important. Images of possible motility may be reinforced by demonstrations of taxis toward nutrients, oxygen, light, or other stimuli, and/or by growth and reproduction (Lawrence *et al.*, 1989; Elfwing *et al.*, 2004). Briefly, living organisms move (a) tens to hundreds of times more *rapidly* than Brownian motion and (b) *meaningfully,* toward or away from stimuli. Imaging can answer fundamental questions about life that chemical techniques cannot. Direct observation of cells directly addresses key aspects of the nature of life: particularly, whether extraterrestrial life is cellular in the way we currently understand it on Earth, and what structure cells assume. Put simply, imaging answers the question “What does extraterrestrial life look like?” With the exception of viruses, all organisms on Earth have been initially identified as being alive by visual observation (macroscopic or microscopic imaging). If organisms in a sample are nonmotile, for example in a biofilm, imaging data may complement chemical data and provide key information supporting the argument of extant life. Even in a biofilm, some organisms at the periphery are likely to be motile. Imaging is not a replacement for chemical biosignature detection—instead it is an important complement.

However, as straightforward as imaging may seem, it is challenging to implement in space or remotely in terrestrial environments. Most microscopy techniques require expert manipulation and are sensitive to vibration and temperature extremes. For these reasons, no direct microscopic observations have been made on Mars with the resolution required to detect bacteria, although microscopes of different kinds have been proposed or accepted for flight. The NRC Committee on an Astrobiology Strategy for the Exploration of Mars wrote, “Atomic force microscopy … is the only way to image in space beyond the limitations of optical devices. Other imaging technologies including interferometry, scanning near-field optical microscopy, and electron microscopy techniques should also be developed for spaceflight applications” (Committee on an Astrobiology Strategy for the Exploration of Mars, 2007). Apart from electron microscopy, progress has been made in developing such instruments for space. Two proposed concepts will be reviewed in detail later in this paper: advanced light microscopy, usually with fluorescence, and holographic (interferometric) microscopy.

## 2. Future Missions Target Liquid Water and Sea Ice

Lander missions have so far been restricted to Mars, which until 2015 (Martin-Torres *et al.*, 2015) was believed to have no liquid water at present at its surface, though it may support liquid water in subsurface regions and probably hosted at least one ocean in the past. Investigations have therefore focused upon the possibility of using morphological traces, as occur in rocks on Earth, to indicate signs of past bacterial motility (Krepski *et al.*, 2013; Rivera and Sumner, 2014). In contrast to past missions, missions over the next several decades will prioritize targets within the Solar System that contain abundant liquid water: Europa (Squyres *et al.*, 1983; Carr *et al.*, 1998), Enceladus (Tsou *et al.*, 2012; Hsu *et al.*, 2015), Titan (Rampelotto, 2012; Turse *et al.*, 2013; McLendon *et al.*, 2015), and Ganymede (McCord *et al.*, 2001; Saur *et al.*, 2015) are all examples.

**FIG. 3.**
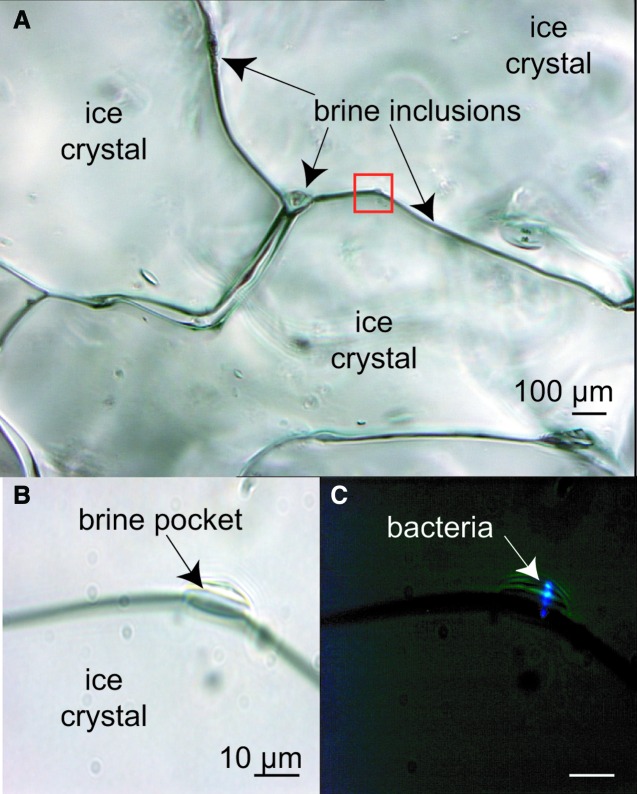
Sea ice that appears solid to the eye and hand is deceptive. Peering into the unmelted ice matrix using light microscopy, on a scale relevant to microorganisms, reveals a network of liquid brine inclusions as thin veins along ice-grain boundaries, at triple-point junctures between ice grains, and in small pockets (visible by transmitted light in the upper and lower left images) that house bacteria. At least some of these bacteria remain metabolically active in these fluids, even as temperatures drop severely during the Arctic winter (Junge *et al.*, 2004). These images were taken at −15°C, when the liquid phase of the ice is approaching its minimum volume. The area outlined in red is (**A**) enlarged in (**B**) and (**C**), where (**C**) was taken by epifluorescence microscopy following staining with the DNA-specific stain DAPI. [Adapted from Junge *et al.* (2001)]

Earth lake ice and sea ice, particularly in high-latitude polar regions, may be the best analogues we have for the layers of ice on the jovian and saturnian moons that are shielded from radiation levels destructive of life as we know it. The composition of the subsurface oceans on the moons is less well constrained. Several lake and sea environments that test the limits of Earth life have been suggested as analogues; limiting factors may be chemical (pH, salinity) or physical (temperature, pressure) (Marion *et al.*, 2003). Although it may appear solid, sea ice is porous with an interior network of microscopic veins and channels filled with brine that contains living microorganisms, including prokaryotes (Fig. 3) and, when sufficient space and nutrients are available, eukaryotes. An analysis of motility in brines and cold oceans on Earth is the first step toward understanding where motility might be expected to be observed elsewhere.

## 3. Hypothesis

The underlying hypothesis of this work is that motile microbes are an inherent feature of natural aquatic habitats, even extreme ones; not all inhabitants may rely upon movement to complete their life histories, but some fraction of the community will have evolved the ability to achieve directed motion via swimming. If not swimming at the time of *in situ* imaging, a controlled shift in some aspect of the environmental conditions (temperature, oxygen, nutrients) can induce or stimulate motility in enough organisms to enable detection. Thus, in any Earth aquatic environment, motility will serve as an unambiguous biosignature.

The limited body of literature on this subject supports this hypothesis, but a significant amount of work remains to be done, and motility is not widely accepted or proposed as a biosignature for missions. We postulate that microbial motility is one of the most likely biosignatures to be found on ocean worlds, one that is definitive and non-Earth-centric. The current state of knowledge is summarized in the next sections.

## 4. Bacterial Motility in Earth's Oceans

The abundance of bacteria in Earth's oceans varies temporally and spatially but typically averages approximately 10^8^ microorganisms per liter, with a lower estimate of 20,000 “species” in each liter (Amaral-Zettler *et al.*, 2010). The majority of marine bacteria in culture are motile, but at any given sampling time in natural environments, the fraction of motile bacteria can be highly variable, with reports ranging from <5% to >80% (Fenchel, 2001; Grossart *et al.*, 2001). Motility levels are higher in the summer than in winter and higher during the day than at night (Grossart *et al.*, 2001). Motile bacteria often respond to chemical stimuli, which may include low-molecular-weight compounds such as single amino acids or more complex organics. Marine bacteria have been shown to exhibit chemotaxis (the ability to sense chemical gradients and bias motility accordingly) toward dissolved amino acids, sugars, carboxylic acids, organic sulfur compounds, oxygen, nitrate, nitrite, ammonium, urea, and phosphate (Segall *et al.*, 1986; Willey and Waterbury, 1989; Yu *et al.*, 1993; ZimmerFaust *et al.*, 1996; Miller *et al.*, 2004; Wadhams and Armitage, 2004; Seymour *et al.*, 2009; Stocker and Seymour, 2012). In cultures of cells displaying poor or no motility, enrichment with tryptic soy broth can cause >80% of the bacteria to become motile within 12 h (Mitchell *et al.*, 1995a).

Motility is not always energetically favorable. Both biological and physical factors determine where and when motility will be favored and what the optimal swimming speed is (Brileya *et al.*, 2013; Di Salvo and Condat, 2014). In seawater, motility is often favored when a high level of dissolved nutrients becomes available, particularly from point sources such as organic particles or individual phytoplankton in an algal bloom, which upon releasing nutrients create a chemical gradient, reinforcing that chemotaxis is one of the fundamental functions of motility. Over the past 20 years, evidence has accrued to confirm that the ocean is not a homogeneous oligotrophic entity but rather a patchy world where micrometer- to millimeter-sized patches of dissolved organic matter represent important sources of nutrients for bacteria (Azam, 1998; Blackburn *et al.*, 1998; Long and Azam, 2001; Stocker, 2012). These patches, which include lysing algae, marine snow particles, fecal pellets, detritus, and sloppy feeding events, have nutrient concentrations 100–10,000 times greater than the picomolar to micromolar concentrations in bulk ambient seawater. Biological factors determining motility include cell size (Mitchell, 1991; Thar and Kuhl, 2003), energetic efficiency of swimming (*e.g.*, flagellar structure) (Macnab and Aizawa, 1984; Magariyama *et al.*, 1994, 2001; Muramoto *et al.*, 1995; Li *et al.*, 2011; Son *et al.*, 2013), and the presence or absence of protozoan predators (Matz and Jurgens, 2005; Wanjugi and Harwood, 2014). Physical factors include the density, size, and lifetime of nutrient patches (Stocker *et al.*, 2008); the presence of fluid flow and in particular turbulence (Luchsinger *et al.*, 1999; Taylor and Stocker, 2012); and the dynamics of swimming at low Reynolds numbers (Purcell, 1977).

A 2002 survey (Johansen *et al.*, 2002) described the motility behavior of 84 cultured strains of marine bacteria. The mean swimming speed measurements for each of these strains ranged from 11.3 to 38.5 μm s^−1^, with a majority of the bacteria having a mean speed of 15–25 μm s^−1^; all these speeds are readily distinguished from Brownian motion by the much faster spatial dispersion (Fig. 2). The study underlined the differences between the motility of these marine strains and that of enteric strains (*e.g.*, *Escherichia coli*) and soil strains (*Bacillus subtilis*). Observations of natural communities of marine bacteria revealed swimming speeds dramatically faster than the speed of *E. coli,* with means of 60–100 μm s^−1^ and bursts up to 400 μm s^−1^ (Mitchell *et al.*, 1995b). Furthermore, the motility pattern of the majority of marine bacteria is starkly different from the “run and tumble” motility of *E. coli;* marine strains show nearly instantaneous reversals of direction. These occur because 90% of motile marine bacterial species have only a single flagellum (monotrichous) (Leifson *et al.*, 1964), as opposed to multiple flagella occurring at random locations on the cell body (peritrichous) as in *E. coli*. Further study led to the recent discovery of a peculiar turning strategy, called the “flick” (Xie *et al.*, 2011), whereby marine bacteria exploit a buckling instability of their (sole) flagellum in order to turn (Son *et al.*, 2013). Taken together, these observations reveal specific adaptations of motility in marine bacteria. Because these adaptations are the result of a typically nutrient-poor environment, where energy conservation for motility is important (Taylor and Stocker, 2012), marine bacteria may represent a useful model system to benchmark algorithms for the detection of motility within samples. These observations have led to detailed physical investigations of the factors that influence swimming in the ocean.

### 4.1. Environmental extremes affecting motility

#### 4.1.1. Nutrient limitation

Taylor and Stocker (2012) modeled marine bacterial motility as an optimal foraging problem. The authors found that, under nutrient conditions comparable to those in coastal regions, rapid bacterial swimming was favored; for conditions more like the oligotrophic open ocean, nonmotile strains were favored.

In an experimental study, Matz and Jurgens (2005) found that carbon (C) limitation favored small, motile bacteria, whereas phosphorus (P) limitation led to dominance of large, elongated, and capsulated bacteria that were not motile. The genotypic community composition did not change, suggesting that the same organisms could display different phenotypes under different conditions. In the presence of eukaryotic flagellate predators, C-limited bacteria displayed increased motility, whereas P-limited bacteria became filamentous. Microscopic observations illustrated the importance of motility in predator avoidance. Strategies to avoid such predation may not be a factor on planets without eukaryotes, although sometimes predators can be other prokaryotes, such as the case of *Bdellovibrio bacteriovorus,* a small bacterium that preys on other bacteria by boring into them and replicating inside their host (Im *et al.*, 2014; Pasternak *et al.*, 2014).

Surface layers of shallow, organic-rich marine sediments, where steep oxygen gradients exist, are habitats for some of the largest and fastest-swimming bacteria. Different organisms show preferences for different specific O_2_ tensions and will swim rapidly into the preferred oxygen concentration band (Fenchel, 1994). Cell sizes up to a striking 25 μm in diameter (*Thiovolum majus*) and swimming speeds in excess of 1000 μm s^−1^ have been observed in these sediments (Fenchel and Thar, 2004). A similar effect might also be observed for other redox couples on worlds without oxygen; the facultative anaerobe *Shewanella oneidensis* MR-1 has been observed to alter its motility in response to the presence of electron shuttles under anaerobic conditions, a phenomenon called electrokinesis that was not described until 2010 (Harris *et al.*, 2010).

Under conditions of extreme energy limitation, motility does not occur (Hoehler and Jorgensen, 2013). This is the likely scenario for deep sub-seabed environments on Earth and for hypothetical extraterrestrial environments that are severely energy-limited. The presence or introduction of localized resources, however, may still favor motility in subpopulations of microbes in an environment considered energy-limited on the bulk scale. These studies have important implications for designing a mission based upon detection of motility, as discussed in the summary section below.

#### 4.1.2. Temperature extremes and ice

**FIG. 4.**
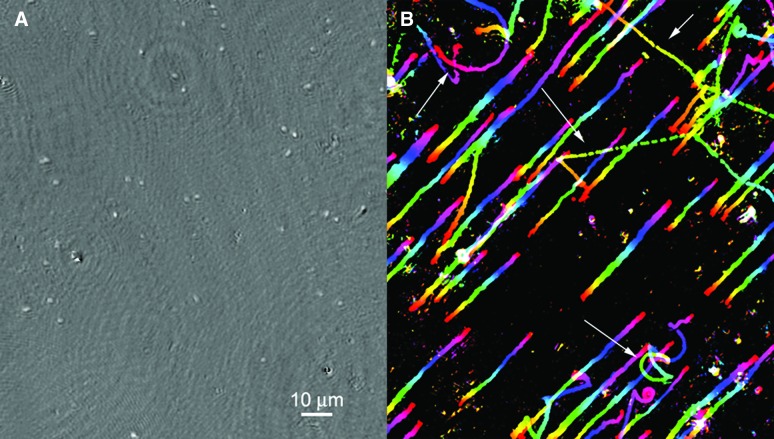
Motility of *Colwellia psychrerythraea* after freezing. (**A**) Appearance of a single *z* plane in phase (Supplementary Video S2). (**B**) Depth-coded tracks showing meaningful swimming (arrows) superimposed upon drift caused by thawing.

The marine bacterium *Colwellia psychrerythraea* strain 34H, a species known to inhabit sea ice as well as cold seawater and sediments, has been shown to be motile at temperatures below −10°C, approaching the limit where hypersaline water freezes (Junge *et al.*, 2003). After freezing for several minutes, this strain will demonstrate immediate motility upon thawing; after freezing for extended periods at −20°C, motility returns over a time course of ∼20 min post-thawing (Fig. 4 and Supplementary Video S2).

**FIG. 5.**
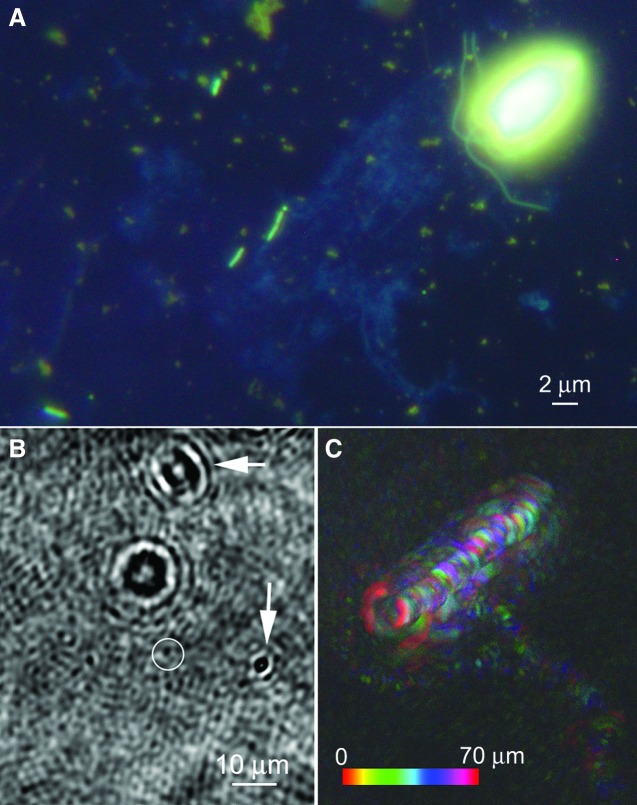
Images of brine from sea ice near Nuuk, Greenland. (**A**) Fluorescence image of acridine orange–stained and DAPI-stained sample showing the prokaryotes that dominate numerically along with a eukaryote; the latter are predominantly photosynthetic diatoms but also include flagellates as shown. (**B**) Reconstructed amplitude image of a hologram of the brine, taken in the field. The image represents a single *z* plane at a single time point. The arrows show nonmotile organisms; the circle identifies a prokaryote clearly identified by its swimming (see Supplementary Video S3). (**C**) Depth-coded swimming trajectories of the motile organisms, which in these panels are one eukaryote (circular swimming pattern) and one prokaryote (apparently chemotaxing toward the eukaryote), in the same experiment as in (B). The color bar indicates depth into the page.

It is not yet known whether this organism displays motility throughout its natural habitats or, more specifically, whether it can swim within the interior brine network of sea ice. Our preliminary data indicate that some motility can be seen immediately after collection of brines from sea ice at temperatures of −3°C to −6°C (Fig. 5 and Supplementary Video S3). Greater recovery of motility may be seen over extended periods of time (days to weeks), a factor that must be considered in mission design (Miteva, 2008).

#### 4.1.3. Cell size

Dusenbery (1997) modeled the effects of cell size on Brownian motion and calculated that motility provided no benefit for cell diameters <0.6 μm, because the effect of rotational Brownian motion overwhelms the directionality in the swimming, making propulsion ineffective in allowing any directional motion. This prediction was consistent with a review of known genera, where motile organisms had cell lengths of >0.8 μm (Dusenbery 1997). Smaller organisms were only found among nonmotile genera. Swimming speed and gradient length determine the minimum cell size at which chemotaxis is meaningful (Mitchell, 1991, 2002). These data provide a useful limit for the design of an imager seeking motility but also suggest that conditions of extreme nutrient limitation could result in populations of very small, nonmotile organisms.

### 4.2. Summary and implications for an astrobiological imager

In Earth's marine environments, even the most extremely cold or oligotrophic, many bacteria are motile or capable of motility (Mykytczuk *et al.*, 2013; Boetius *et al.*, 2015; Lindensmith *et al.*, 2016). Physical arguments and direct observations suggest that a minimum cell size of 0.6–0.8 μm is a requirement for motility, as is the presence of phosphate. Exposure of nonmotile cells to rich broth or point sources of nutrients often causes motility to manifest within minutes to hours. These data help set the parameters of an instrument designed to look for motility in an extraterrestrial lander. Spatial resolution should be on the order of the smallest expected cells but does not need to be significantly better, as motility may be observed in subresolved images through image analysis (*e.g.*, through image differences). Imaging may be performed immediately, but if motility is not observed, then nutrients could be introduced and allowed to incubate with samples for at least 12 h to stimulate swimming. Such nutrients may be simple, low-molecular-weight molecules such as ammonia, glucose, or amino acids. Both chiralities should be included in a test instrument unless a preferred chirality is established in the target before the mission.

On icy worlds, it may not be necessary to access subsurface oceans, as life—including organisms capable of motility—is present throughout Earth's icy environments (Boetius *et al.*, 2015). Further studies in Earth analog environments, including aqueous environments with extremes in pH and oxygen availability (Glein *et al.*, 2015), are needed to determine when and how motility may best be used as a biosignature.

Some environments on Earth do not appear to host motile bacteria, particularly deep subseafloor sediments. If life-detection missions to similar environments are planned, it will be important to determine whether long-term treatments to stimulate motility are sufficient for use of motility as a biosignature. If this is not possible, imaging will need to focus on the ability to detect organisms (including submicrometer-sized prokaryotes) by morphology. This places greater demands upon the imaging instrument, as discussed below.

## 5. Mission Implementation

**FIG. 6.**
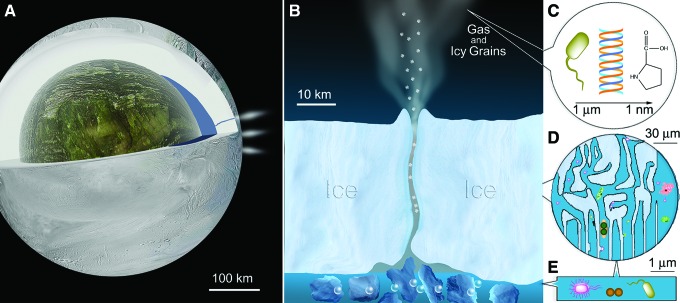
Sampling strategies for Enceladus. (**A**) The moon is 504 km in diameter, with an ice crust containing numerous areas of cryovolcanic activity. (**B**) The area around a single cryovolcano provides several options for sampling. (**C**) A flyby may sample the plumes directly; survival of molecules in samples depends upon the capture energy and may represent anything from whole bacterial cells or spores (useful for imaging) to simple compounds (which must be detected spectroscopically). (**D**) The surface ice is likely to contain fissures, which could harbor life as observed on Earth. (**E**) Sampling the subsurface ocean would give access to free-living organisms. (Image credits: NASA/JPL for panels A and B)

There are at least three conceivable sources of samples on icy moons: ejected fluids captured in a flyby, samples from surface ice, and samples from subsurface oceans (Fig. 6). The following sections discuss each of these.

### 5.1. Flyby

At least one, and possibly as many as three, of the moons of Jupiter and Saturn are ejecting subsurface fluids into space. A flyby mission could capture ejecta without the energetic cost and complexity of a lander, and the sample could be investigated remotely by instruments in the spacecraft or returned to Earth. This approach is likely to yield the smallest volume of all collection methods; depending on the mission design, it might be as small as microscopic ice crystals in aerogel, as achieved by the Stardust mission. Multiple flybys would be necessary to collect enough sample volume for definitive life detection, by any means (Tsou *et al.*, 2012). The sample would be collected at a high speed; particles captured by the Cassini spacecraft impacted at 23,000 to 63,000 km h^−1^ (6.4–17.5 km s^−1^) (Srama *et al.*, 2004; Hsu *et al.*, 2010, 2011). Cassini speeds would at most preserve small molecules such as amino acids (Leroux and Jacob, 2013), which would be detected spectroscopically; an imager would be of little use under these conditions. Based upon panspermia studies, if collection speeds could be reduced to 1.5–5 km s^−1^, viable bacteria and spores could be preserved (Burchell *et al.*, 2001, 2003, 2010, 2014a, 2014b). In this implementation, a microscopic imager could be flown in the spacecraft to “prescreen” samples for morphological indications of cells or spores. This prescreening would not provide definitive life detection itself but could be used to improve the chances of definitive life detection by using a combination of additional techniques. The presence or absence of cell-like structures seen by the imager could help determine the number of flybys to perform or influence the choice of samples to return to Earth for more detailed analysis. If such a mission concept is considered, the sampling process and suitable collection volumes would need to be investigated in the context of preserving the equivalent of bacterial cells or spores. Such studies have not yet been performed.

### 5.2. Surface ice

**FIG. 7.**
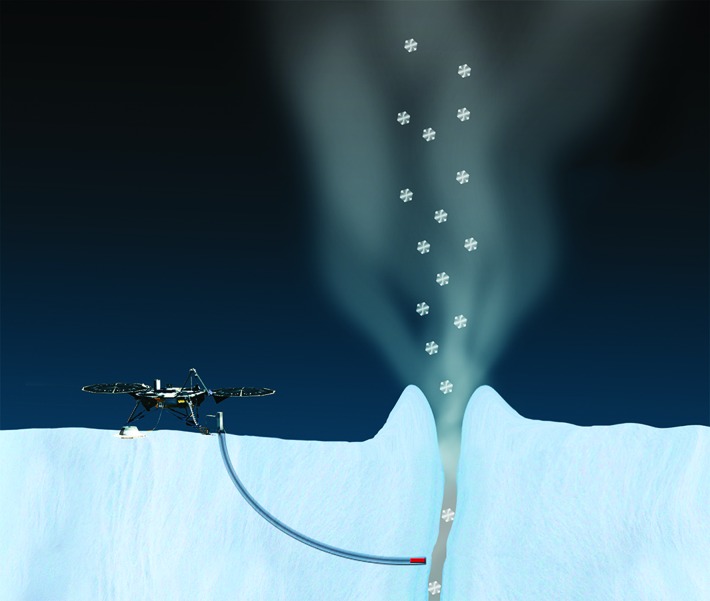
Artist's concept of IceMole sampling strategy. The probe melts through the ice to encounter water near a plume, while the rover remains at a safe distance. [Concept: Enceladus Explorer (EnEx) consortium/German Space Agency (DLR)]

While sample return, even from a small moon, is extremely challenging, a lander would allow for sophisticated analysis of surface samples on site. All the candidate moons have icy surfaces, and flight instruments have been developed for sampling a scoop or core and moving it as solid or liquid into analytical systems. Several Mars landers have featured scoops for sampling soil from the planet's surface (Perko, 2007). The Philae comet lander on the ESA Rosetta mission was equipped with a corer that could drill more than 20 cm into the surface, collect samples, and deliver them to different ovens or for microscopic inspection (Schulz, 2010). Targets for surface ice investigation include Mars, Europa, Enceladus, Ganymede, and others. Concepts have been presented for an Enceladus lander that combine drilling (or screwing) with melting in a way such that the direction of the probe can be controlled. Called IceMole, the probe has been tested in glaciers on Earth and shown to be controllable in three dimensions and able to sample without forward contamination (Razzaghi *et al.*, 2008; Konstantinidis *et al.*, 2015) (Fig. 7).

If we use the *Europa Study 2012 Report* (Europa Study Team, 2015) as an example starting point, it might be possible to incorporate a microscope for observation of motility or structure with only small modification to the baseline design. The study report lander provides a ∼1 mL sample of ice to two or more small sample chambers that are then sealed and heated to vaporize the sample and drive it into the spectrometers. To observe possible microbes, the sample would be initially heated only enough to melt the ice into water, and possibly a few degrees above the melting point. The micro-imager could be configured to look directly into the large sample chamber, or possibly a constricted region (*e.g.*, 1 mm^3^ for a digital holographic microscope) that is optimized for the microscope's performance. This would give a reasonable chance of direct observation of microbes present at concentrations of ∼10^4^ mL^−1^. To search for microbes at lower concentrations, some mechanism would be required to slowly feed the whole melted sample (probably less than 1 mL after melting unless the ice is from a solid sample) through the sample chamber observed by the microscope. To avoid additional mechanisms on the lander, simple gas pressure might be used by slightly heating the sample chamber to increase the vapor pressure. A full milliliter could be driven through a 1 mm^3^ sample chamber in less than an hour at rates that would allow images of any particle or cell-like objects that pass through. Data volume could become an issue in screening such a large volume for motility, so some image processing would be required on board to evaluate images or sequences that should be stored and transmitted (*e.g.*, due to sufficient frame-to-frame change). After the sample passes through the microscopic imager, it can then be passed to the high-temperature chamber for vaporization and spectroscopic analysis. Data from the spectrometers can then be correlated to the micro-imager to provide independent observations of the same sample.

### 5.3. Subsurface oceans

Liquid samples from drilling down to subsurface oceans may be the most desirable targets, but they are the most difficult to obtain, especially on Europa. A significant amount of technology development has attempted to address the problem of accessing an ocean under several kilometers of ice. Earth analogues exist in the form of subglacial Antarctic lakes, which have been accessed by drilling; forward contamination is a serious concern, as are the mass and power requirements of the drill (Bulat *et al.*, 2011; Blythe *et al.*, 2014; Burnett *et al.*, 2014; Rack *et al.*, 2014). Other groups have proposed smaller penetrators for the europan surface that would sample several centimeters of surface ice but not penetrate the ice crust (Biele *et al.*, 2011; Weiss *et al.*, 2011). Radiation is expected to be prohibitive for eukaryotes in the first ∼10 cm of Europa's crust, though bacteria can be more resistant. At 1 mm the radiation dose is 1 Gy min^−1^, which the bacterium *Deinococcus radiodurans* can tolerate without effects on growth rate. At 10 cm the dose rate is 5 mGy min^−1^, which most Earth bacteria can survive (Baumstark-Khan and Facius, 2002).

**FIG. 8.**
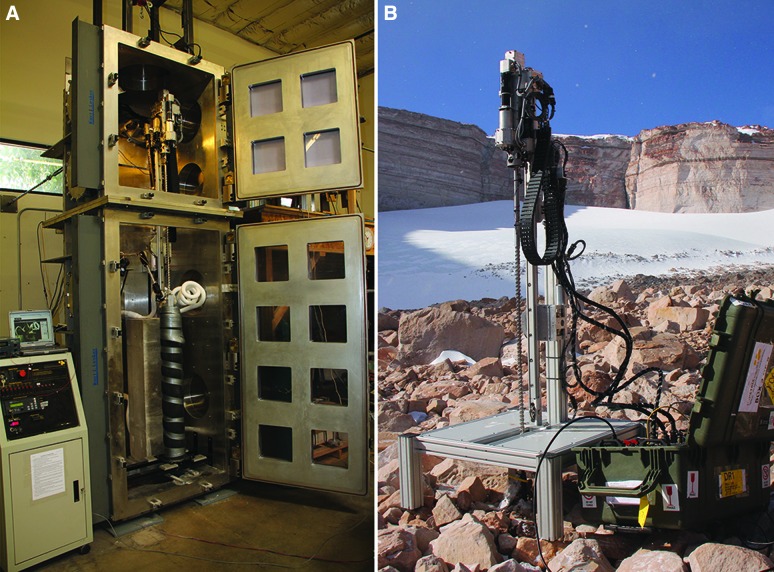
Drill developed for sampling deep ice on Mars. (**A**) In the laboratory. (**B**) Deployed in the Antarctic Dry Valleys. (Images courtesy of Honeybee Robotics)

Other targets may be more readily achievable. The Enceladus ocean is under at least 25 km of ice (Olgin *et al.*, 2011; Patthoff and Kattenhorn, 2011) but may be as little as 1 m below the surface of the ice in the area of the “tiger stripes,” so it may be accessed by currently available technology, such as the drill developed for the Mars Icebreaker mission (Glass *et al.*, 2011; Zacny *et al.*, 2013) (Fig. 8). The astrodynamics of landing on small bodies is well understood (Ulamec and Biele, 2009; Ulamec *et al.*, 2014). The imager would be located on the lander vehicle and data relayed to an orbiter. While collection of the sample might be different from a surface lander, processing of the sample would be very similar.

Table 1 presents a notional science traceability matrix starting from the specific science questions that can be addressed by microscopy (left-most column) and progressing to preliminary instrument and sample-handling requirements. The matrix shows a number of indicators of life that are accessible to microscopy but that cannot be found via strictly chemical observations. The performance requirements and goals are based on size ranges for terrestrial archaea and bacteria, which are reasonable starting points for potential extant life in liquid oceans elsewhere in the Solar System. The parameters most likely to drive instrument design and selection are the sample volumes required and the processing needed to provide a sample that meets the conditions for effective imaging by the particular type of microscope selected. The next section describes a number of microscopy approaches and their constraints for potential life-detection missions.

**Table 1.** Science Traceability Matrix for a Microscopic Imager

*Specific science goal*	*Observable*	*Performance requirement*	*Performance goal*	*Sample conditions*	*Volumes required*
Detect cell-like objects	Microscopic image	0.8 μm resolution (motile terrestrial prokaryotes); limit of detection 10^4^ cells mL^−1^ (10× more dilute than seawater) determined by *xyz* field of view	0.3 μm (nonmotile terrestrial prokaryotes); limit of detection 100 cells mL^−1^	On slide/suspension in liquid	∼0.25 μL at 10^4^ mL^−1^ density; ∼25 mL at 100 mL^−1^ density at minimum performance requirement
Distinguish density of different objects	Measure Brownian motion	Velocity resolution ∼100 μm s^−1^		Suspension in liquid; known temperature; viscosity measurement (useful not required)	Sufficient to have particles present
Detect structure in objects	Microscopic image	0.8 μm resolution	0.3 μm	Instrument dependent	Instrument dependent
Test for life	Detect motility of cell-like objects	Velocity resolution 2 μ s^−1^, dynamic range up to ∼500 μ s^−1^ motion/frame < ∼0.1 frame/frame	Velocity resolution <1 μ s^−1^, frame rate >10 fps		
	Feeding/Culture of cell-like objects	Evaporate 1 mL of sample, heat-sterilize, mix new culture in			
	Chemotaxis	Supply inorganic agents			
	Phototaxis	3 additional light sources (LED)	Varying wavelength sources		
	Magnetotaxis	Electromagnet ∼2× ambient field of planet			
	Detect specific cell components	Specific dye labeling with dyes for membrane, nucleic acid, protein OR downstream coupling to mass spectrometry	Additional panel of dyes targeting cell wall components, esterases, etc.	On slide with dyes added	Pre-concentrated sample of at least 1 mL (at 10^4^ mL^−1^ concentrations) or 100 mL (for 100 mL^−1^ concentrations)

The overall NASA goal is “Search for/detect life on Ocean Worlds or Mars.”

## 6. Instrument Concepts

There are several challenges facing any microscope for space missions: spaceflight robustness; ability to operate autonomously, especially with regard to focus; and ability to span the wide spatial scales across which unicellular life may exist. Modern microscopes are heavy and contain fragile components, particularly in the optical train. Compound objectives contain multiple lenses that are extremely sensitive to alignment and may not survive the vibrations of launch. As a result of these factors, microscopic imagers that have been selected for flight have had significantly scaled-down optical components. The most sophisticated microscope intended for Mars landing was on board the lost Beagle 2. The microscope consisted of a Cook triplet lens with 40 μm depth of focus coupled to a fixed-focus monochromatic camera. Illumination was provided by LEDs of four colors [wavelengths for red, green, blue, and UVA (373 nm)] (Thomas *et al.*, 2004). The entire microscope was mechanically translated along its axis in increments of the depth of field in order to focus; in-focus elements were then combined into an image. Improved algorithms for such image processing have since been developed (Luethi *et al.*, 2010). The resolution was approximately 4 μm pixel^−1^. This instrument has been used on Earth to investigate mineralized biosignatures (Pullan *et al.*, 2008). It was proposed in revised form for the MicrOMEGA instrument on board ExoMars but was cut during descoping. It has since been coupled with a laser ablation mass spectrometer to produce a multimodal instrument called CAMAM (Tulej *et al.*, 2014). Other types of instruments with similar spatial resolution have also been proposed, some spanning several orders of spatial resolution, from centimeters to millimeters (Fink *et al.*, 2013; Nunez *et al.*, 2014). While this type of spatial resolution is ideal for mineralogy and perhaps fossilized microbial biosignatures (Hofmann, 2008; Pullan *et al.*, 2008), it is insufficient for observation of living microorganisms and not at all intended for time-lapse imaging of motile specimens. Several emerging instrument concepts have been proposed to directly target micrometer-scale cells. The use of morphology as a sole biosignature imposes additional requirements, as discussed below.

### 6.1. High-performance light microscopes

A light microscopy module consisting of a modified commercial high-performance light microscope is found on the International Space Station. The instrument is based upon a commercial off-the-shelf (COTS) Leica RXA microscope, modified to be operated remotely. Several commercial objectives are available. Sample chambers are investigator-specific for both biological and materials applications; electric field and temperature control are future upgrades. This instrument is expected to be upgraded for confocal performance in 2015–2016.

**FIG. 9.**
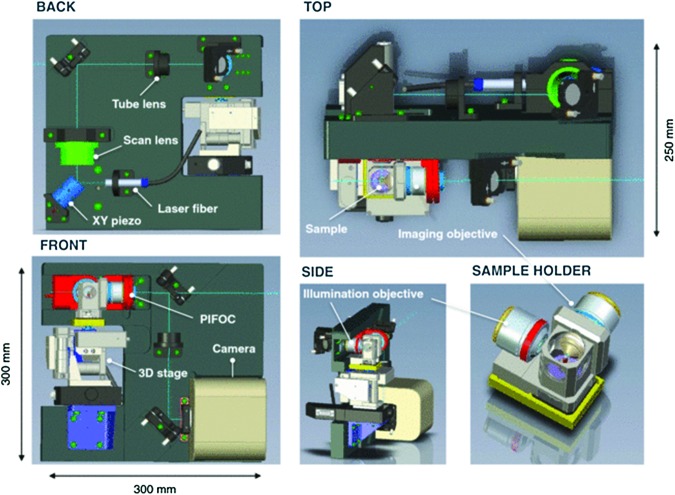
Laser scanning microscope concept for biological space research. The model shows an orthogonal illumination setup including a sample holder, visualized from different angles. [Image from De Vos *et al.* (2014) *Rev Sci Instrum* 85, doi:10.1063/1.4898123, reproduced with permission of the American Institute of Physics]

The General Support Technology Program of ESA funded the development of a compact confocal laser scanning microscope (Beghuin *et al.*, 2005; De Vos *et al.*, 2014) (Fig. 9). Its confocal mode has excitation at 488 nm, and there is also the capability for fluorescence lifetime imaging (FLIM) with excitation at 630 nm and time resolution of 200 ps. The same hardware permits operation in fluorescence recovery after photobleaching (FRAP) and fluorescence loss in photobleaching (FLIP) modes. Brightfield [differential imaging contrast (DIC)] is also supported and may be acquired simultaneously with fluorescence. The optics are COTS Zeiss components. While this type of design is conceived for detailed cell studies on the International Space Station, it might be used as a tool in an orbiter to observe samples such as those that may be obtained from Enceladus’ plumes. A method for automatic focusing would need to be established in this case.

The biggest drawback to high-resolution light microscopy for life detection is throughput volume required to locate an individual entity. The minimum objective magnification that permits bacterial imaging is approximately 40 × , which gives a depth of field of ∼2 μm. A 2k × 2k CCD with 3 μm pixel^−1^ gives a field of view at 40 × of ∼(0.3 mm)^2^, so that each image samples a volume of 1.8 × 10^−4^ μL. Therefore, for a microbial concentration of 10^5^ to 10^6^ cells mL^−1^, which is typical of Earth's surface ocean, this sampled volume represents significantly less than an average of one cell per image. Preconcentration of samples is thus necessary for almost all environmental samples when using the technique of light microscopy.

**FIG. 10.**
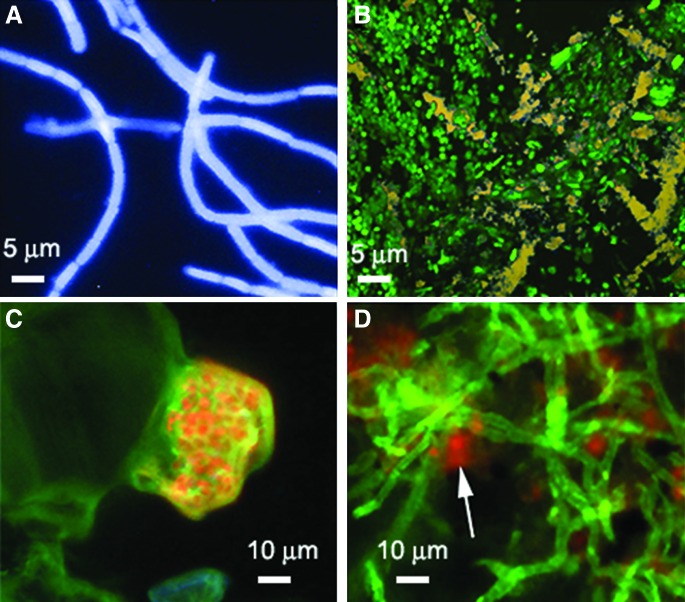
Detection of microorganisms within environmental samples by fluorescent dye labeling. (**A**) *Bacillus subtilis* in soil labeled with violet-fluorescent wheat germ agglutinin Gram-positive-specific cell wall stain. (**B**) Arctic biofilm (predominantly *Thiomicrospira*) stained with SYTO-9, a bacterial stain for nucleic acids. The green is the SYTO-9; the yellow matrix represents sulfur minerals. (**C**) Microorganisms in a gypsum endolith labeled with carboxyfluorescein diacetate, a probe for esterase activity that does not label dead cells. (**D**) Green-fluorescent wheat germ agglutinin labeling fungal hyphae in a Mojave Desert endolith. The red fluorescence is chlorophyll autofluorescence from photosynthetic cyanobacteria within this microbial community.

The use of morphology as a biosignature places additional requirements on sample preparation that pose challenges for space. Confocal laser scanning microscopy and its derivatives such as FLIM, FRAP, and FLIP require samples to be fluorescent. Fluorescent labeling with dyes, followed by high-resolution light microscopic imaging, is the best tool for identification of nonmotile cells and for use of morphology as a biosignature for two key reasons: fluorescence imaging increases the specific signal relative to the background, and it permits identification of subresolved structures such as flagella, cell membranes, and nucleoids. Ultraresolution labeling techniques can improve spatial resolution to tens of nanometers (Huang *et al.*, 2010). Fluorescence microscopy is the current standard on Earth for enumeration of organisms in environmental samples including water, soil, and rock (Porter and Feig, 1980; Kepner and Pratt, 1994; Kawasaki 1999) (Fig. 10). The most commonly used dyes are the nucleic acid probes DAPI and acridine orange, which label nucleic acids; other fluorescent dyes are available to target a variety of biosignature compounds such as lipids and cell walls (Fig. 10A–10C).

We have previously discussed in depth the feasibility of using fluorescent dye labeling as a tool for life detection (Nadeau *et al.*, 2008). In principle, it is both feasible and promising, although labeling in extreme environments is a challenge because dyes are sensitive to heat and cold as well as to radiation (both light and ionizing). There are also planetary protection concerns associated with bringing complex organic molecules to astrobiological targets, an issue which may exclude most dyes. Additionally, effective staining techniques require substantial sample processing that would add complexity to a space instrument. An alternative is to make use of microbial autofluorescence. All organisms show autofluorescence with ultraviolet excitation, although the spectra are nonspecific and the emission is usually weak relative to that of dyes; the required excitation wavelengths (260–280 nm) are difficult to obtain in flight-qualified instruments. Some pigments, such as chlorophyll, absorb and emit strongly in the visible (Fig. 10D). Although it cannot be expected that extraterrestrial organisms would necessarily evolve pigments with spectra similar to those found on Earth, organisms within our solar system may show similar spectra of photosynthetic and photoprotective pigments because they evolved with the same sun (Kiang *et al.*, 2007; Schwieterman *et al.*, 2015).

Despite these concerns, fluorescence imaging with dye labeling remains the best technique for definitive identification of submicrometer-scale structures with the ability to distinguish biosignature molecules. It should be the technique of choice for any environment where motility is unlikely. If dye labeling is not possible, the parameters of a fluorescence microscope intended for life detection should be carefully chosen to permit observation of the most common types of autofluorescence. Any life detection based upon morphology should be coupled to spectroscopic techniques that will confirm the presence of the biomolecules putatively imaged (lipids, amino acids, nucleic acids). These techniques are well developed, and interfacing them to an imager may in many cases be as straightforward as introducing a beamsplitter.

While fluorescence microscopy can also detect motility, other emerging techniques are potentially superior for flight because they obviate the need for the complex optics, preconcentration, and dyes, as discussed in the following section.

### 6.2. Digital holographic microscopy (DHM)

**FIG. 11.**
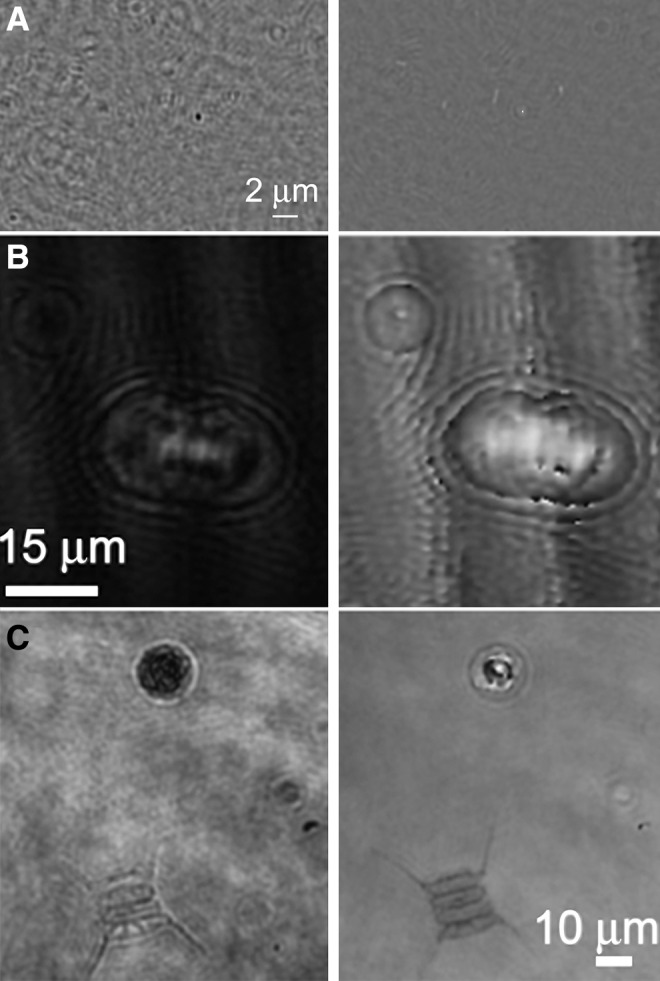
Amplitude (left) and phase (right) images from holographic images of (**A**) a culture of the bacterium *Escherichia coli;* (**B**) cultured *Paramecium micromultinucleatum;* and (**C**) an untreated sample of pond water showing a photosynthetic algal cell (round) and a diatom.

Holography is a well-established imaging technique that uses the interference of light to record and reproduce 3-D images of objects. Its use in optical applications began in the 1960s with the invention of the laser (Knox, 1966). DHM has several advantages over ordinary imaging microscopy that make it ideal for field and astrobiology use, including no need for focus or scanning so that instruments are readily made autonomous. DHM can produce simultaneous bright-field and quantitative phase-contrast images of the same field (Fig. 11) (Mann *et al.*, 2005; Marquet *et al.*, 2005), providing additional information about transparent objects, for example, refractive index and/or thickness; thus, it inherently supports effective label-free imaging. There are two major approaches to DHM: using incoherent light and using coherent light (lasers).

#### 6.2.1. DHM with incoherent light

**FIG. 12.**
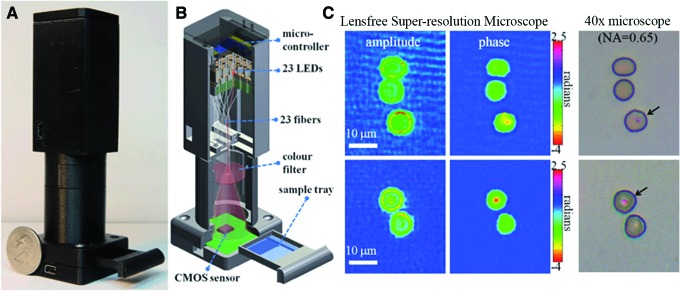
Photograph (**A**) and schematic (**B**) of a lens-free super-resolution microscope. Twenty-three individual multimode fiber-optic cables are butt-coupled to 23 LEDs without the use of opto-mechanical components. Each LED is turned on sequentially and used as an illumination source to create lens-free holograms of the objects on a complementary metal oxide semiconductor sensor array. (**C**) Imaging of red blood cells infected with malaria parasites (*Plasmodium falciparum*) in a standard thin blood smear, compared with comparable images taken with a 40× objective (NA 0.65) on a bright-field microscope. Republished with permission of the Royal Society of Chemistry from Bishara *et al.* (2011).

Digital holographic microscopy based upon LEDs or an array of LEDs is a notable approach to lensless microscopy that can yield extremely small, robust, low-power instruments. These LEDs may be coupled to microfluidics for complete on-chip systems. Mudanyali *et al.* (2010) described an instrument weighing <50 g that achieved 1–2 μm resolution over a field of view of 24 mm^2^ (approximately 10-fold that of a 10 × objective). A single amber (591 nm) LED was used for illumination. By placing two thin polarizers and two thin birefringent crystals in front of the sensor array, the authors demonstrated differential interference contrast imaging with the same instrument. Bishara *et al.* (2011) used an array of 23 red (633 nm) LEDs that were sequentially illuminated in order to improve resolution. The image generated with each LED is a slightly shifted hologram, allowing for use of a pixel super-resolution algorithm to achieve ∼800 nm spatial resolution. The field of view is 24 mm^2^ without scanning, and the entire instrument weighs <100 g (Fig. 12).

The advantages of this approach are very low mass and low power, insensitivity to alignment, and lack of mechanically sensitive optics. The drawbacks are low depth of field, so that 3-D tracking of motility is not possible, and corresponding lack of throughput sample volume similar to what is seen with traditional light microscopy. This type of instrument has not yet been tested in an astrobiological context; it may be a suitable backup instrument for situations where very low mass and power are required. However, for optimal detection of motility, observation of the full 3-D sample depth is preferable, which requires a design based upon coherent light illumination.

#### 6.2.2. DHM with coherent light

Digital holographic microscopes with laser illumination have been developed for laboratory and field use. One commercial instrument was flown in parabolic flight to demonstrate performance in low gravity (Toy *et al.*, 2012). The advantage of coherent DHM is that it has a large depth of field, because the entire volume contributes to the recorded hologram. Digital refocusing of samples can typically be achieved on 50 × the classical depth of field at full resolution, and particle tracking at lower resolution is possible over an even greater depth. Not only does this approach provide quantitative 3-D records of motility, it also allows for greater throughput than any other microscopy technique able to probe up to milliliters of fluid at a time. Limits of detection for bacteria are 10^6^ mL^−1^ or better. We have reported an off-axis design with 800 nm lateral resolution, ∼1 μm depth resolution, and the ability to track bacteria of different swimming speeds within a sample volume of ∼0.6 × 0.4 × 0.4 mm^3^ per image (Wallace *et al.*, 2015).

**FIG. 13.**
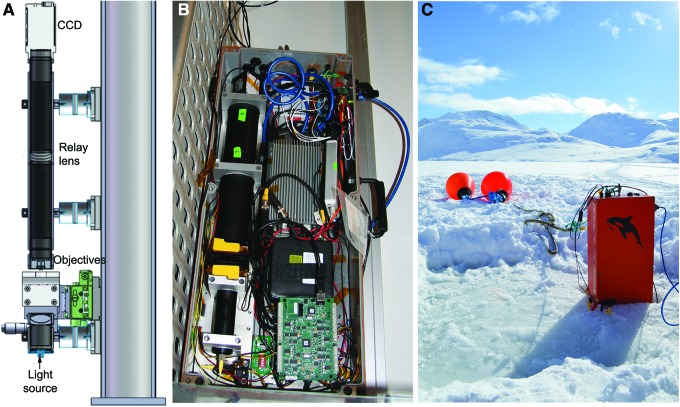
Two-beam holographic microscope for field use. (**A**) Schematic of the shared optical path design, as described by Wallace *et al.* (2015). (**B**) A fieldable version containing onboard computer and electronics. (**C**) Deployed in a sackhole for examination of sea ice brines in Kobbefjord, Greenland (this instrument and setup were used in the collection of the data for Fig. 5 in this paper).

A fully fieldable version of a similar design has recently been developed. This instrument has a shared optical path for the object and reference beams, making most disturbances common mode and the interference fringes inherently stable. The parallel-axis design is also very insensitive to misalignment, which simplifies manufacture and minimizes mass. The field instrument weighs 10 kg including COTS packaging and electronics and an onboard computer (Fig. 13). The mass of the optical system (including laser and COTS 4 Mpx camera) is <1 kg. Power consumed by the laser and camera is <6 W, with total system power for the field instrument <15 W. This instrument was successfully used to detect motility in Greenland sea ice brines, demonstrating a limit of detection of ∼10^4^ organisms per milliliter without sample preconcentration. Eukaryotic cells were identified unambiguously by morphology and motility. While capable of resolving prokaryotes, the microscope did not provide sufficient morphological detail to definitively identify prokaryotic cells as alive unless they were motile (Lindensmith *et al.*, 2016). Therefore, this type of instrument is so far primarily of use for detecting motility. DHM instruments are diffraction-limited in the same way as imaging microscopes, and further refinement is possible to achieve higher resolution, though possibly at the expense of larger optics and higher mass. It may also be possible to develop specific dyes for holography that will permit visualization of subcellular structures.

## 7. Conclusion

Although meaningful motion is an unambiguous biosignature, it has not been well explored for astrobiology for two primary reasons: uncertainty as to its universality, and lack of instrumentation for its detection on the microbial scale. Recent developments have addressed both issues. Studies of microbial motility on Earth have set limits on the minimum size for motile microorganisms and the conditions under which bacteria express motility or not. Improvements in instrumentation have also led to several viable designs for microscopic imagers that can be used for spaceflight. Interferometric microscopy provides high throughput but lower spatial resolution and is better for observing motility; advanced light microscopy is superior for imaging cell structures, especially in combination with dyes. Both imaging modalities might be combined into an instrumentation suite, which should also contain instruments for confirmation of chemical biosignatures such as lipids, nucleic acids, and amino acids.

## Supplementary Material

Supplemental data

Supplemental data

Supplemental data

## Abbreviations Used

COTS: commercial off-the-shelf
DHM: digital holographic microscopy
FLIM: fluorescence lifetime imaging
FLIP: fluorescence loss in photobleaching
FRAP: fluorescence recovery after photobleaching
