# Supplementary material for: Microbial Morphology and Motility as Biosignatures for Outer Planet Missions
Source: Astrobiology. 2016 Oct 1;16(10):755–74. doi: 10.1089/ast.2015.1376 (PMC5069736; doi:10.1089/ast.2015.1376)
Supplement: Supplemental data [file Supp_Video1.zip › Supp_Video1.pdf]

Supplementary Data

SUPPLEMENTARY VIDEO S1. A phase-contrast image (20× objective) of a liquid sample containing both passive particles (1 μm beads) and motile microorganisms (*Vibrio alginolyticus* bacteria). Under video microscopy, passive particles showing Brownian motion can easily be distinguished from motile microorganisms.
