# Supplementary material for: Microbial Morphology and Motility as Biosignatures for Outer Planet Missions
Source: Astrobiology. 2016 Oct 1;16(10):755–74. doi: 10.1089/ast.2015.1376 (PMC5069736; doi:10.1089/ast.2015.1376)
Supplement: Supplemental data [file Supp_Video2.zip › Supp_Video2.pdf]

SUPPLEMENTARY VIDEO S2. Motility of *Colwellia psychrerythraea* after freezing. Shown is a single *z* plane in phase at 15 frames/s. Meaningful swimming is superimposed upon drift caused by thawing.
