# Supplementary material for: Microbial Morphology and Motility as Biosignatures for Outer Planet Missions
Source: Astrobiology. 2016 Oct 1;16(10):755–74. doi: 10.1089/ast.2015.1376 (PMC5069736; doi:10.1089/ast.2015.1376)
Supplement: Supplemental data [file Supp_Video3.zip › Supp_Video3.pdf]

SUPPLEMENTARY VIDEO S3. Reconstructed amplitude image of a hologram of Greenland sea ice brine, taken in the field. The video represents a single z plane imaged at 15 frames/s. Some larger, nonmotile organisms are seen along with a prokaryote clearly identified by its swimming.
